# Fuzzy Reasoning Numerical Spiking Neural P Systems for Induction Motor Fault Diagnosis

**DOI:** 10.3390/e24101385

**Published:** 2022-09-28

**Authors:** Xiu Yin, Xiyu Liu, Minghe Sun, Jianping Dong, Gexiang Zhang

**Affiliations:** 1 Academy of Management Science, Business School, Shandong Normal University, Jinan 250014, China; 2 College of Business, The University of Texas at San Antonio, San Antonio, TX 78249, USA; 3 Research Center for Artificial Intelligence, Chengdu University of Technology, Chengdu 610059, China; 4 School of Automation, Chengdu University of Information Technology, Chengdu 610225, China

**Keywords:** fuzzy reasoning numerical spiking neural P systems, interval-valued triangular fuzzy numbers, fault diagnosis

## Abstract

The fuzzy reasoning numerical spiking neural P systems (FRNSN P systems) are proposed by introducing the interval-valued triangular fuzzy numbers into the numerical spiking neural P systems (NSN P systems). The NSN P systems were applied to the SAT problem and the FRNSN P systems were applied to induction motor fault diagnosis. The FRNSN P system can easily model fuzzy production rules for motor faults and perform fuzzy reasoning. To perform the inference process, a FRNSN P reasoning algorithm was designed. During inference, the interval-valued triangular fuzzy numbers were used to characterize the incomplete and uncertain motor fault information. The relative preference relationship was used to estimate the severity of various faults, so as to warn and repair the motors in time when minor faults occur. The results of the case studies showed that the FRNSN P reasoning algorithm can successfully diagnose single and multiple induction motor faults and has certain advantages over other existing methods.

## 1. Introduction

Induction motors are widely used to drive various mechanical and industrial equipment. The major components of an induction motor are usually stators, rotors, air gaps and bearings [1,2]. Due to their heavy workload and harsh working environment, induction motors are prone to various hidden troubles during operations. The occurrence of faults usually causes huge economic losses, so it is necessary to detect faults early, prevent the occurrence and development of faults and prevent the occurrence of destructive and catastrophic accidents [3,4,5]. The fault diagnosis of induction motors generally consists of two processes including state detection and diagnosis. Specifically, by monitoring and analyzing its relevant operating parameters, the current operating state of an induction motor is evaluated to determine whether a fault exists. If it is in a fault state, the location, severity and development trend of the fault need to be clarified [6,7].

In recent years, motor fault diagnosis methods based on artificial neural networks [8,9,10,11] have become a research hotspot. Mejia-Barron et al. [12] proposed a multi-layer neural network-based model to reproduce the current characteristics associated with inter-turn short circuit fault conditions, providing a new tool for testing and monitoring the induction motor working conditions. Deng et al. [11] proposed a new method for bearing fault diagnosis based on empirical wavelet transform, fuzzy entropy and support vector machines. Kumar and Hati [6] proposed a new detection technique for bearing faults and broken rotor bars of squirrel-cage induction motors based on an extended convolutional neural network model. Although neural networks can be used to find solutions according to the faults that need to be resolved, they also have obvious disadvantages, such as the need to learn from a large number of samples, slow convergence and serious local optimal solutions [13]. In addition, the above-mentioned methods do not have the ability to detect faults in complex conditions and cannot conduct a comprehensive diagnosis of the entire machine [14,15].

As a new high-performance distributed parallel computing model, fuzzy reasoning spiking neural P systems (FRSN P systems) [16] have been widely used in power system fault diagnosis and have achieved good results. Since spiking neural P systems (SN P systems) do not have the ability to deal with fuzzy and uncertain data in fault diagnosis problems, the FRSN P systems integrate different fuzzy logics into SN P systems. Various fuzzy reasoning algorithms for fault diagnosis using FRSN P systems have been developed [15,16,17,18,19,20,21]. An SN P system consists of a network of neurons connected together with synapses and can be regarded as the third generation of neural network models. SN P systems transmit information with pulses (spikes) among neurons through synapses [22]. A SN P system is also a directed graph, in which nodes represent neurons, and the connections between nodes represent synapses. Spikes are transmitted from presynaptic neurons to postsynaptic neurons along the synapses [23]. Variants of SN P systems have been developed and have been applied not only to power system fault diagnosis but also to Boolean circuits modeling [24], combinatorial optimization [25], image processing [26,27] and fingerprint recognition [28].

Fault diagnosis using FRSN P systems stems from the similarity between the transmission of impulses between neurons through synapses and the propagation of faults in power systems [4,29]. Although FRSN P systems have been used for fault diagnosis of power systems, spikes are only used as a “tool” in FRSN P systems to perform operations between the values that spikes can represent, i.e., real numbers in the interval [0, 1]. In order to take full advantage of numerical operations, this work adopts the numerical spiking neural P system (NSN P system) proposed by Wu et al. [30] and uses it for fault diagnosis of induction motors. The NSN P systems establish the connection between SN P systems and NP systems by replacing the spikes and the evolution rules in the SN P systems with numerical variables and programs in the NP systems, respectively, but still retaining the directed graph structure of the SN P systems.

The NSN P system is used first to solve the Boolean satisfiability (SAT) problem, a NP-complete problem, to demonstrate its computational capability, before the FRNSN P system is used for motor fault diagnosis. The SAT problem and the motor fault diagnosis problem have certain similarities since their cores are propositional formulas. Therefore, the NSN P system is capable of solving the motor fault diagnosis problem if it can successfully solve the SAT problem.

In order to better characterize the uncertainty in motor fault diagnosis, the fuzzy reasoning numerical spiking neural P systems (FRNSN P systems) are developed by introducing the interval-valued triangular fuzzy numbers (IVTFNs) [31] into NSN P systems in this study. The FRNSN P reasoning algorithm is designed based on the operating mechanism of the FRNSN P system. The FRNSN P systems are used to model the faults, and the FRNSN P reasoning algorithm is used to diagnose the faults of the induction motors. In addition, the relative preference relationship is used to estimate the severity of various potential faults of the motors in order to detect the faults in a timely manner. The contributions of this work are summarized as follows:

1. The NSN P system, as a combination of the SNP system and the NP system, is applied to motor fault diagnosis for the first time. In order to prove its ability to deal with induction motor fault diagnosis, the NSN P system is used to solve the SAT problem first. The results show that the NSN P system can successfully solve the SAT problem in six steps;
2. The IVTFNs are applied to the NSN P system, and the FRNSN P system is proposed to deal with the incompleteness and uncertainty of motor fault information. The FRNSN P system can successfully model the fault fuzzy production rules of induction motors;
3. A FRNSN P reasoning algorithm is designed by using the operating mechanism of FRNSN P systems, making the motor fault diagnosis intelligent;
4. The relative preference relationship is used to estimate the severity of multiple faults when they occur, so as to diagnose the faults in a timely manner and to prevent the deterioration of the faults.

The rest of this paper is organized as follows. Section 2 provides preliminaries on the IVTFNs and the relative preference relations. Section 3 presents the NSN P systems, shows a computational example, and gives the definition of the FRNSN P systems. Section 4 describes the fuzzy reasoning process of the FRNSN P systems and designs the FRNSN P reasoning algorithm. Section 5 reports the computational results to show the effectiveness of the FRNSN P reasoning algorithm for fault diagnosis of induction motors.

## 2. Preliminaries

### 2.1. The Interval-Valued Triangular Fuzzy Number

**Figure 1:**
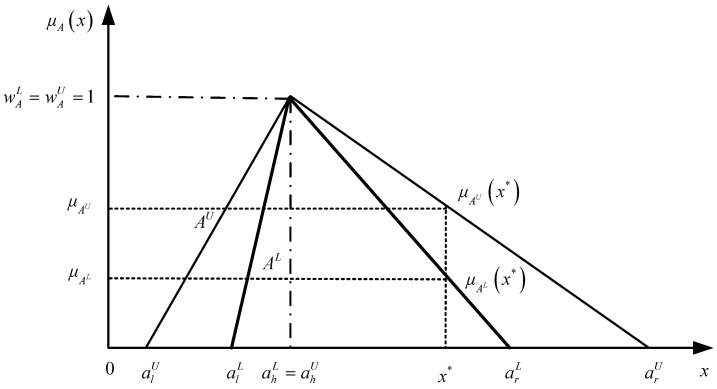
An NIVTFN A.

An IVTFN is defined as $A=\left[A^{L}A^{U}\right]=\left[\left(a_{l}^{L},a_{h}^{L},a_{r}^{L};w_{A}^{L}\right),\left(a_{l}^{U},a_{h}^{U},a_{r}^{U};w_{A}^{U}\right)\right]$, where $A^{L}$ and $A^{U}$ represent the lower and upper limits of A and $A^{L}\subseteq A^{U}$. When $w_{A}^{L}=w_{A}^{U}=1$ and $a_{h}^{L}=a_{h}^{U}$, the form of A becomes $A=\left[A^{L},A^{U}\right]=\left[\left(a_{l}^{U},a_{l}^{L}\right),\left(a_{h}^{L}=a_{h}^{U}\right),\left(a_{r}^{L},a_{r}^{U}\right)\right]=\left[\left(a_{l}^{U},a_{l}^{L}\right),a_{h},\left(a_{r}^{L},a_{r}^{U}\right)\right]$, which is called a normalized IVTFN (NIVTFN). An NIVTFN is shown in Figure 1, where $\mu_{A}\left(x\right)$ is the membership function representing the degree of the membership of x, and $\mu_{A^{L}}\left(x\right)$ and $\mu_{A^{U}}\left(x\right)$ are the lower and the upper bounds of $\mu_{A}\left(x\right)$ [31].

### 2.2. The Relative Preference Relation

Suppose $\Psi =\left\{A_{1},A_{2},\ldots ,A_{n}\right\}$ is a set of n IVTFNs, with $A_{i}=[(a_{il}^{U},a_{il}^{L}),a_{ih},(a_{ir}^{L},a_{ir}^{U})]$ for i=1,2,…,n. The average of $A_{i}$ is represented by $\bar{A}$ given by $\bar{A}=\left[\left({\bar{a}}_{l}^{U},{\bar{a}}_{l}^{L}\right),{\bar{a}}_{h},\left({\bar{a}}_{r}^{L},{\bar{a}}_{r}^{U}\right)\right]$. A membership function $\mu_{\beta }\left(A_{i},\bar{A}\right)\in \left[0,1\right]$ with a relative preference relation β expresses the preference of $A_{i}$ to $\bar{A}$. A $\mu_{\beta }\left(A_{i},\bar{A}\right)<\frac{1}{2}$ means $\bar{A}$ takes precedence over $A_{i}$, and a $\mu_{\beta }\left(A_{i},\bar{A}\right)>\frac{1}{2}$ means $A_{i}$ takes precedence over $\bar{A}$. The membership function $\mu_{\beta }\left(A_{i},\bar{A}\right)$ is defined in (1) as follows

(1) $$\;\mu_{\beta }\left(A_{i},\bar{A}\right)=\frac{1}{2}\left(p\times \frac{\left(a_{il}^{L}-{\bar{a}}_{r}^{L}\right)+2\left(a_{ih}-{\bar{a}}_{h}\right)+\left(a_{ir}^{L}-{\bar{a}}_{l}^{L}\right)}{2\times \left\|T_{S}^{L_{+}},T_{S}^{L_{-}}\right\|}+\left(1-p\right)\times \frac{\left(a_{il}^{U}-{\bar{a}}_{r}^{U}\right)+2\left(a_{ih}-{\bar{a}}_{h}\right)+\left(a_{ir}^{U}-{\bar{a}}_{l}^{U}\right)}{2\times \left\|T_{S}^{U_{+}},T_{S}^{U_{-}}\right\|}\right)\;for\;0\leq p\leq 1,$$

where $\left\|T_{S}^{L+},T_{S}^{L-}\right\|=\{\begin{array}{ll} \frac{\left(t_{sl}^{L+}-t_{sr}^{L-}\right)+2\left(t_{sh}^{+}-t_{sh}^{-}\right)+\left(t_{sr}^{L+}-t_{sl}^{L-}\right)}{2}, & \mathrm{if}\;t_{sl}^{L+}\geq t_{sr}^{L-}\\ \frac{\left(t_{sl}^{L+}-t_{sr}^{L-}\right)+2\left(t_{sh}^{+}-t_{sh}^{-}\right)+\left(t_{sr}^{L+}-t_{sl}^{L-}\right)}{2}+2\left(t_{sr}^{L-}-t_{sl}^{L+}\right), & \mathrm{if}\;t_{sl}^{L+}<t_{sr}^{L-} \end{array}$, $\left\|T_{S}^{U+},T_{S}^{U-}\right\|=\{\begin{array}{ll} \frac{\left(t_{sl}^{U+}-t_{sr}^{U-}\right)+2\left(t_{sh}^{+}-t_{sh}^{-}\right)+\left(t_{sr}^{U+}-t_{sl}^{U-}\right)}{2}, & \mathrm{if}\;t_{sl}^{U+}\geq t_{sr}^{U-}\\ \frac{\left(t_{sl}^{U+}-t_{sr}^{U-}\right)+2\left(t_{sh}^{+}-t_{sh}^{-}\right)+\left(t_{sr}^{U+}-t_{sl}^{U-}\right)}{2}+2\left(t_{sr}^{U-}-t_{sl}^{U+}\right), & \mathrm{if}\;t_{sl}^{U+}<t_{sr}^{U-},\;\mathrm{and} \end{array}$ $\begin{array}{l} T_{S}^{L-}=\left(t_{sl}^{L-},t_{sh}^{-},t_{sr}^{L-}\right)=\left(\min\left\{a_{il}^{L}\right\},\min\left\{a_{ih}\right\},\min\left\{a_{ir}^{L}\right\}\right)\\ T_{S}^{L+}=\left(t_{sl}^{L+},t_{sh}^{+},t_{sr}^{L+}\right)=\left(\max\left\{a_{il}^{L}\right\},\max\left\{a_{ih}\right\},\max\left\{a_{ir}^{L}\right\}\right)\\ T_{S}^{U-}=\left(t_{sl}^{U-},t_{sh}^{-},t_{sr}^{U-}\right)=\left(\min\left\{a_{il}^{U}\right\},\min\left\{a_{ih}\right\},\min\left\{a_{ir}^{U}\right\}\right)\\ T_{S}^{U+}=\left(t_{sl}^{U+},t_{sh}^{+},t_{sr}^{U+}\right)=\left(\max\left\{a_{il}^{U}\right\},\max\left\{a_{ih}\right\},\max\left\{a_{ir}^{U}\right\}\right) \end{array}$, for i=1,2,…,n.

In the above relative preference relationship, the coefficients p and 1−p are the weights of the lower interval $A^{L}$ and the upper interval $A^{U}$, respectively. The value of p, called the relative preference relation value, is generally determined subjectively, and several different values are usually considered. A good relative preference relationship has a value of p close to 1, and a poor relative preference relationship has a value of p close to 0. Therefore, the relative merits of IVTFNs in a specific set can be quickly judged by the relative preference relation value p [13,31,32].

## 3. The NSN P System and Its Extension to the FRNSN P System

The NSN P systems are described and their computational power is demonstrated by solving SAT problems. The FRNSN P system is then defined by introducing the IVTFNs into the NSN P system, which lays the foundation for fault diagnosis of induction motors.

### 3.1. The NSN P System

The NSN P system, described in detail below, has a slightly different threshold from that used in the literature [30,33].

An NSN P system is defined as a tuple as shown in (2) below:

(2) $$\prod =(\sigma_{1},\sigma_{2},\ldots ,\sigma_{l},syn,in,out),$$

where l≥1 is the degree of the NSN P system. The notations in this definition are given below.

(1) $\sigma_{1},\sigma_{2},\ldots ,\sigma_{l}$ represent *l* neurons with the form $\sigma_{k}=\left(\theta_{k},Var_{k},{\mathrm{Pr}}_{k},Var_{k}\left(0\right)\right)$, for 1≤k≤l, where
  (a) $\theta_{k}\in \mathbb{Z}$ is the threshold of neuron $\sigma_{k}$;
  (b) $Var_{k}=\left\{x_{w,k}|1\leq w\leq h_{k}\right\}$ is a set of variables in neuron $\sigma_{k}$, where *h_k_* is the number of variables in $\sigma_{k}$;
  (c) $Var_{k}\left(0\right)=\left\{x_{w,k}\left(0\right)|x_{w,k}\left(0\right)\in \mathbb{R},1\leq w\leq h_{k}\right\}$ refers to the set of initial values of the variables in the set $Var_{k}$;
  (d) ${\mathrm{Pr}}_{k}=\left\{pr_{Ρ,k}=F_{Ρ,k}\left(x_{1,k},\ldots ,x_{h_{k},k}\right)|1\leq Ρ\leq {h^{\prime }}_{k}\right\}$ is a set of programs, where F is called a production function in neuron $\sigma_{k}$, where ${h^{\prime }}_{k}$ is the number of programs in $\sigma_{k}$.
(2) syn={(k,j)|1≤k,j≤l,k≠j} is the set of synapses.
(3) in and out correspond to the input neuron $\sigma_{in}$ and the output neuron $\sigma_{out}$, respectively.

In NSN P system Π, $x_{w,k}$ and $pr_{Ρ,k}$ represent variable w and program Ρ in neuron $\sigma_{k}$, respectively. When neuron $\sigma_{k}$ has only one variable or only one program, w or Ρ is omitted from the subscripts. At time t, the value of variable $x_{w,k}$ is represented by $x_{w,i}\left(t\right)$ and the production value of program $pr_{Ρ,k}$ is represented by $pr_{Ρ,k}\left(t\right)=F_{Ρ,k}\left(x_{1,k}\left(t\right),\ldots ,x_{h_{k},k}\left(t\right)\right)$, i.e., the production value $pr_{Ρ,k}\left(t\right)$ is determined by the values of the variables $x_{1,k},\ldots ,x_{h_{k},k}$ at time t. Each neuron in Π has a threshold $\theta_{k}$, and program $pr_{Ρ,k}$ will be applied only when $pr_{Ρ,k}\left(t\right)\geq \theta_{k}$. Once $pr_{Ρ,k}$ is applied, meaning neuron $\sigma_{k}$ fires, the values of the variables $x_{1,k},\ldots ,x_{h_{k},k}$ are reset to 0 and $pr_{Ρ,i}\left(t\right)$ is simultaneously transmitted to the variables of the postsynaptic neurons of neurons $\sigma_{k}$. If $pr_{Ρ,k}\left(t\right)<\theta_{k}$, neuron $\sigma_{k}$ will not fire and $pr_{Ρ,k}\left(t\right)$ will disappear at this moment.

If the sum of the production values received by variable $x_{w,k}$ at time t is *pr*(*t*), then $x_{w,k}(t+1)$ is updated according to (3) in the following:

(3) $$x_{w,k}(t+1)=\{\begin{array}{ll} pr(t), & \mathrm{if}\;\mathrm{the}\;\mathrm{application}\;\mathrm{of}\;\mathrm{program}\;pr_{Ρ,k}\;\mathrm{involves}\;\mathrm{variable}\;x_{w,k}\\ pr(t)+x_{w,k}(t), & \mathrm{if}\;\mathrm{the}\;\mathrm{application}\;\mathrm{of}\;\mathrm{program}\;pr_{Ρ,k}\;\mathrm{does}\;\mathrm{not}\;\mathrm{involve}\;\mathrm{variable}\;x_{w,k} \end{array}$$

All neurons work in parallel in the NSN P system, and each neuron applies one program at most at each moment. If more than one program can be applied, only one can be selected non-deterministically.

### 3.2. An Application to the SAT Problem

A SAT problem checks whether the variables of a given Boolean formula can be consistently replaced with the values TRUE and FALSE such that the formula evaluates to TRUE. The instances of SAT problems are determined by two parameters m and n representing the numbers of clauses and variables, respectively. Given a set of Boolean variables $\mathbb{Q}=\left\{q_{1},q_{2},\ldots ,q_{n}\right\}$, a clause C can be expressed in the form $q_{1}\left(\neg q_{1}\right)\vee \cdots \vee q_{i}\left(\neg q_{i}\right)\vee \cdots \vee q_{n}\left(\neg q_{n}\right)$, where ∨ indicates the disjunction. A $q_{i}=1$ means that $q_{i}$ is assigned a true value. In general, if $q_{i}=1$, then $\neg q_{i}=0$, and if $q_{i}=0$, then $\neg q_{i}=1$. As long as a variable in C is given a true value, C is assigned a value of 1, meaning C is satisfiable. The SAT problem is stated as:

INSTANCE: A clause set $\mathbb{C}=\left\{C_{1},C_{2},\ldots ,C_{m}\right\}$, constructed from a finite set $\left\{q_{1},q_{2},\ldots ,q_{n}\right\}$ of Boolean variables.

TASK: Find if there is an assignment of the variables $q_{1},q_{2},\ldots ,q_{n}$ satisfying all the clauses in ℂ.

When the assignment of the variables satisfies all the clauses, ℂ is satisfiable and each clause $C_{j}$, for 1≤j≤m, is given a value of 1. In the following, the SAT problems are solved uniformly with a family of NSN P systems.

**Figure 2:**
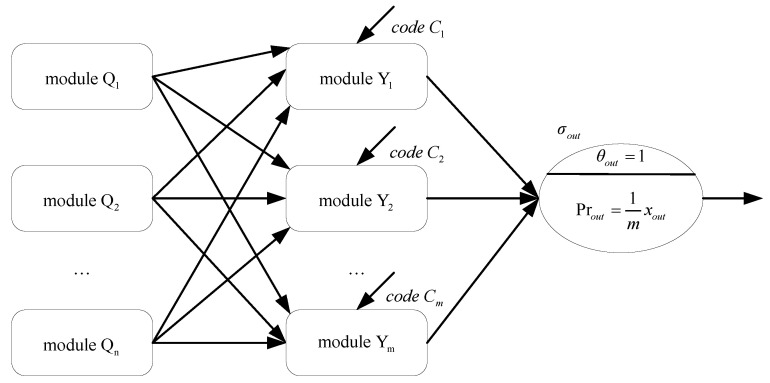
The structure of the NSN P systems for solving the SAT problems.

The NSN P systems, working non-deterministically, can solve the SAT problem in finite time steps. The general structure of the NSN P systems is shown in Figure 2, with modules $Q_{i}$, for 1≤i≤n, and $Y_{j}$, for 1≤j≤m, corresponding to variables $q_{i}$ and clauses $C_{j}$, respectively. Each module $Q_{i}$ has three synapses connected to module $Y_{j}$.

The following method is used to encode a given SAT instance in order to obtain a uniform solution. A propositional formula $\gamma =C_{1}\wedge C_{2}\wedge \ldots \wedge C_{m}$ is considered in the conjunctive normal form, where ∧ indicates the conjunction. Since variable $q_{i}$ may or may not appear in a clause $C_{j}$ and can or cannot be negated when it appears, two bits binary numbers are used to code the relationship between $q_{i}$ and $C_{j}$ with 00 indicating $q_{i}$ not appearing in $C_{j}$, 01 or equivalently 10 indicating $q_{i}$ appearing in $C_{j}$, and 11 indicating $\neg q_{i}$ appearing in $C_{j}$.

Each clause corresponds to an input neuron, and a sequence of 2n digits of 0 s and 1 s is introduced into the input neuron to describe the clause. Therefore, 2n steps are required to input the code of the clause with n variables. For example, $\gamma =\left(\neg q_{1}\vee q_{2}\right)\wedge \left(q_{1}\vee \neg q_{3}\right)$ is a propositional formula composed of clauses $C_{1}=\neg q_{1}\vee q_{2}$ and $C_{2}=q_{1}\vee \neg q_{3}$, and the sequences 110100 and 010011 corresponding to clauses $C_{1}$ and $C_{2}$ will be introduced into the associated input neurons within six steps, respectively.

**Figure 3:**
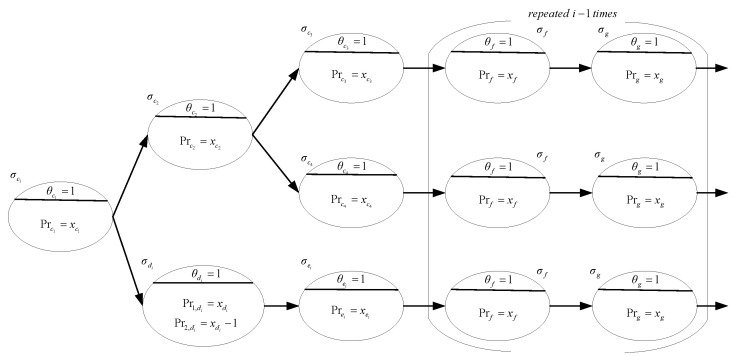
Module $Q_{i}$.

Module $Q_{i}$ is shown in Figure 3. The neurons $\sigma_{c_{1}}$, $\sigma_{c_{2}}$, $\sigma_{c_{3}}$ and $\sigma_{c_{4}}$ in each module $Q_{i}$ are allowed to appear only once in order to reduce the computational complexity. Initially only variable $x_{c_{1}}$ of neuron $\sigma_{c_{1}}$ is assigned a value of 1. Module $Q_{i}$ non-deterministically produces a truth assignment for variable $q_{i}$ by non-deterministically choosing a program between ${\mathrm{Pr}}_{1,d_{i}}=x_{d_{i}}$ and ${\mathrm{Pr}}_{2,d_{i}}=x_{d_{i}}-1$ in neuron $\sigma_{d_{i}}$. Neuron $\sigma_{e_{i}}$ will fire if program ${\mathrm{Pr}}_{1,d_{i}}=x_{d_{i}}$ is applied and will not fire if program ${\mathrm{Pr}}_{2,d_{i}}=x_{d_{i}}-1$ is applied. In this way, neuron $\sigma_{e_{i}}$ transmits the value of 1 or nothing to neuron $\sigma_{z_{j}}$ in module $Y_{j}$. Then $q_{i}$ is assigned the true value if the value of 1 is transmitted. In addition to feeding neuron $\sigma_{d_{i}}$, neuron $\sigma_{c_{1}}$ initially transmits a value of 1 to neuron $\sigma_{c_{2}}$. This value is transmitted along the path $\sigma_{c_{3}}\to \sigma_{f}\to \cdots \to \sigma_{g}$ or $\sigma_{c_{4}}\to \sigma_{f}\to \cdots \to \sigma_{g}$ to neuron $\sigma_{z_{j}}$ in module $Y_{j}$.

**Figure 4:**
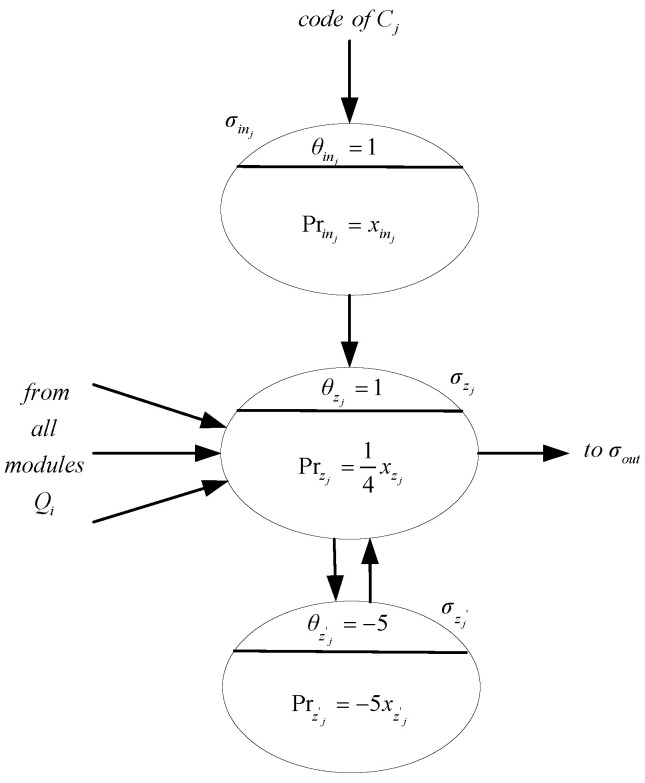
Module $Y_{j}$.

Delay neurons labeled $\sigma_{f}$ and $\sigma_{g}$ are used to maintain the synchronization of the transmission, i.e., neuron $\sigma_{z_{j}}$ receives the value from module $Q_{i}$ and the value from the input neuron associated with clause $C_{j}$ simultaneously. For example, module $Q_{1}$ does not need delay neurons and module $Q_{2}$ needs two delay neurons per row. By analogy, each row of module $Q_{i}$ needs i−1 pairs of delay neurons to guarantee synchronization. Therefore, in step 1+2i, neuron $\sigma_{z_{j}}$ receives the assignment of variable $q_{i}$ and the value from the input neuron. Further processing will be carried out in module $Y_{j}$, as shown in Figure 4.

In steps 3, 5, …, 2n+1, neuron $\sigma_{z_{j}}$ may receive the following values:

2 if $q_{i}=0$, but $q_{i}$ and $\neg q_{i}$ do not appear in $C_{j}$,
3 if $q_{i}=1$, but $q_{i}$ and $\neg q_{i}$ do not appear in $C_{j}$,
3 if $q_{i}=0$, but $q_{i}$ appears and $\neg q_{i}$ does not appear in $C_{j}$,
4 if $q_{i}=1$, but $q_{i}$ appears and $\neg q_{i}$ does not appear in $C_{j}$,
4 if $q_{i}=0$, but $\neg q_{i}$ appears and $q_{i}$ does not appear in $C_{j}$,
5 if $q_{i}=1$, but $\neg q_{i}$ appears and $q_{i}$ does not appear in $C_{j}$.

Program ${\mathrm{Pr}}_{z}=\frac{1}{4}x_{z}$ in neuron $\sigma_{z_{j}}$ will be activated and will produce a value of 1 in two cases, one is when $q_{i}=1$ and $q_{i}$ appears in $C_{j}$ and the other is when $q_{i}=0$ and $\neg q_{i}$ appears in $C_{j}$. In either case, the assignment of variable $q_{i}$ satisfies clause $C_{j}$. Neuron $\sigma_{{z^{\prime }}_{j}}$ is used to ensure that $\sigma_{z_{j}}$ fires only once by passing the production value −5 to variable $x_{z_{j}}$. In this way, it also ensures that variable $x_{out}$ receives a value of 1 at most once.

In step 2i+2, if all clauses are satisfied, the sum of the values received by variable $x_{out}$ is m, and neuron $\sigma_{out}$ fires. So far, it shows that there is a variable assignment so that the proposition formula γ is satisfiable. Therefore, NSN P systems, containing a total of $6n^{2}-n+2m+1$ neurons working non-deterministically, can solve the SAT problem in finite time steps.

**Figure 5:**
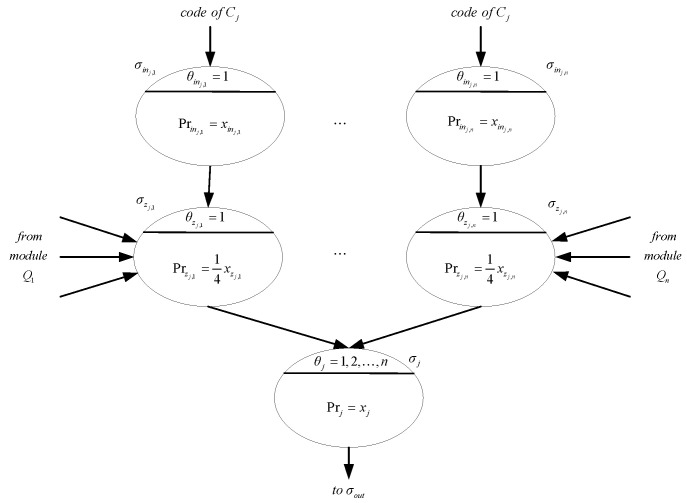
Modified module $Y_{j}$.

The computation time can be shortened by using more input neurons after modifying modules *Y_j_*, j=1,2,…,m. The structure of the modified module $Y_{j}$ is shown in Figure 5. The modified module $Y_{j}$ uses n input neurons to introduce the binary code of a clause in two steps instead of bit by bit in one input neuron. Each of these n neurons receives a two bit binary number 00, 01 (or 10) or 11. When receiving a value of 1, the input neuron $\sigma_{in_{j,i}}$ will fire and transmit a value of 1 to neurons $\sigma_{z_{j,i}}$, 1≤i≤n and 1≤j≤m. Neurons $\sigma_{z_{j,i}}$ and $\sigma_{z}$ in module $Y_{j}$ have similar structures and perform the same functions, i.e., checking whether the assignment of variable $q_{i}$ satisfies clause $C_{j}$.

The delay neurons $\sigma_{f}$ and $\sigma_{g}$ in module $Q_{i}$ are no longer needed while all other parts remain unchanged. In step 3, module $Q_{i}$, for 1≤i≤n, also transmits values of 2 or 3 to all neurons $\sigma_{z_{j,i}}$. All variables and all clauses are then checked in parallel. In step 4, if the assignment of variable $q_{i}$ satisfies clause $C_{j}$, the program in neuron $\sigma_{z_{j,i}}$ will be enabled. As a result, variable $x_{j}$ of neuron $\sigma_{j}$ may receive values of 1,2,…,n. No matter which value $\sigma_{j}$ receives, program ${\mathrm{Pr}}_{1,j}=x_{j}$ will be enabled in step 5 due to the values of the threshold $\theta_{j}=1,2,\ldots ,n$.

The firing of neuron $\sigma_{j}$ shows that $C_{j}$ is satisfiable. Each neuron $\sigma_{j}$ is connected to neuron $\sigma_{out}$. In step 6, if the sum of the values received by neuron $\sigma_{out}$ is m, the assignments of variables $q_{1},q_{2},\ldots ,q_{n}$ all satisfy the clauses in ℂ. At the same time, program ${\mathrm{Pr}}_{out}=\frac{1}{m}x_{out}$ is applied and neuron $\sigma_{out}$ fires. Obviously, the SAT problem is solved in six steps. With the modified modules $Y_{j}$, the NSN P systems use a total of 2nm+m+2n+5 neurons. The computation time is greatly reduced compared to the system with the original modules $Y_{j}$ although it is not clear about how many more neurons are needed.

To show the computational power of the NSN P system, its time steps for solving the SAT problem are compared with those of DDSN P systems [34], WSN P systems [35] and SN P systems with neuron division and budding [36]. The comparisons are shown in Table 1. Obviously, the NSN P system can solve the SAT problem with the fewest steps.

### 3.3. Definition of the FRNSN P System

The FRNSN P system is presented in this subsection as an extension of the NSN P system. A fuzzy reasoning numerical spiking neural P (FRNSN P) system of degree m is defined in (4) as follows

(4) Π=(γ,syn,in,out)

where $\gamma =\gamma_{p}\cup \gamma_{r}=\left\{\sigma_{1},\ldots ,\sigma_{l}\right\}$ is a set of l neurons, with $\gamma_{p}=\left\{\sigma_{1},\ldots ,\sigma_{s}\right\}$ representing the set of proposition neurons and $\gamma_{r}=\left\{\sigma_{s+1},\ldots ,\sigma_{s+n}\right\}$ representing the set of rule neurons, such that l=s+n. Each proposition neuron has the form of $\sigma_{i}=\left(\theta_{i},x_{i},{\mathrm{Pr}}_{i},x_{i}\left(0\right)\right)$, for 1≤i≤s, and each rule neuron has the form of $\sigma_{j}=\left(\theta_{j},c_{j},x_{j},{\mathrm{Pr}}_{j},x_{j}\left(0\right)\right)$, for 1≤j≤n. The details of the notations in the definition of Π are given below.

(1) 
  (a) $\theta_{k}\in \Psi$ is the firing threshold of neuron $\sigma_{k}$, for 1≤k≤l;
  (b) $c_{j}\in \Psi$ indicates the confidence factor of neuron $\sigma_{j}$, for 1≤j≤n.
  (c) $x_{k}$ is the variable of neuron $\sigma_{k}$, for 1≤k≤l;
  (d) $x_{k}\left(0\right)$ is the initial fuzzy value of variable $x_{k}$, for 1≤k≤l.
  (e) ${\mathrm{Pr}}_{k}=\{pr_{k}=F_{k}\left(x_{k}\right)=\{\begin{array}{ll} x_{k}, & \mathrm{if}\;\mathrm{neuron}\;\sigma_{k}\;\mathrm{is}\;a\;\mathrm{propositinal}\;\mathrm{neuron}\\ x_{k}c_{k}, & \mathrm{if}\;\mathrm{neuron}\;\sigma_{k}\;\mathrm{is}\;a\;\mathrm{rule}\;\mathrm{neuron} \end{array}$, is a set of programs, where F is called the production function, for 1≤k≤l.
(2) syn⊆{1,2,…,l}×{1,2,…,l} with (k,k)∉syn is the set of synapses.
(3) in and out correspond to the input neuron $\sigma_{in}$ and the output neuron $\sigma_{out}$, respectively.

In the FRNSN P system, each neuron contains only one variable and one program, each threshold $\theta_{k}$, each confidence factor $c_{j}$ or the initial value of the variable $x_{k}$ is an NIVTFN and each program has only two special forms $pr_{k}=x_{k}$ and $pr_{k}=x_{k}c_{j}$. Everything else in the FRNSN P system is the same as that in the NSN P system.

For convenience and intuition, NIVTFNs are associated with some linguistic semantics. The linguistic semantics used in this work are widely used in the literature [13,15,31] and are shown in Table 2. These linguistic semantics vividly reflect the probability that an event occurs.

In addition, the following arithmetic and logic operations, involved in the operations of the FRNSN P system, are defined.

Premise: $A=\left[\left(a_{l}^{U},a_{l}^{L}\right),a_{h},\left(a_{r}^{L},a_{r}^{U}\right)\right]$ and $B=\left[\left(b_{l}^{U},b_{l}^{L}\right),b_{h},\left(b_{r}^{L},b_{r}^{U}\right)\right]$ are two NIVTFNs, with a and b being real numbers in the interval [0, 1].

Given the above premise, the following arithmetic operation is defined:

$$A\times B=\left[\left(a_{l}^{U}\times b_{l}^{U},a_{l}^{L}\times b_{l}^{L}\right),a_{h}\times b_{h},\left(a_{r}^{L}\times b_{r}^{L},a_{r}^{U}\times b_{r}^{U}\right)\right]$$

Given the above premise, the following logical operations are defined:

(1) And: $A\wedge B=\left[\left(a_{l}^{U}\wedge b_{l}^{U},a_{l}^{L}\wedge b_{l}^{L}\right),a_{h}\wedge b_{h},\left(a_{r}^{L}\wedge b_{r}^{L},a_{r}^{U}\wedge b_{r}^{U}\right)\right]$, where a∧b=min(a,b);
(2) Or: $A\vee B=\left[\left(a_{l}^{U}\vee b_{l}^{U},a_{l}^{L}\vee b_{l}^{L}\right),a_{h}\vee b_{h},\left(a_{r}^{L}\vee b_{r}^{L},a_{r}^{U}\vee b_{r}^{U}\right)\right]$, where a∨b=max(a,b);
(3) If a≥b, then A≥B.

## 4. The FRNSN P Reasoning Algorithm

This section first uses the FRNSN P system to model the fuzzy production rules of the induction motors, and then proposes the FRNSN P reasoning algorithm based on the reasoning process of the FRNSN P system.

### 4.1. Modeling and Fuzzy Reasoning

Fuzzy production rules are usually used for knowledge representation, and the following three types of fuzzy production rules are involved in this work:

*General rule*$R_{j}$*:* IF $p_{1}$, THEN $p_{2}$ $\left(C=c_{j}\right)$;
*And rule*$R_{j}$*:* IF $p_{1}$ AND $p_{2}$ AND … AND $p_{s-1}$, THEN $p_{s}$ $\left(C=c_{j}\right)$;
*Or rule*$R_{j}$*:* IF $p_{1}$ OR $p_{2}$ OR … OR $p_{s-1}$, THEN $p_{s}$$\left(C=c_{j}\right)$;

where $p_{1},\ldots ,p_{s}$ are fuzzy propositions, and $C=c_{j}$ represents the credibility of the fuzzy production rule $R_{j}$.

**Figure 6:**
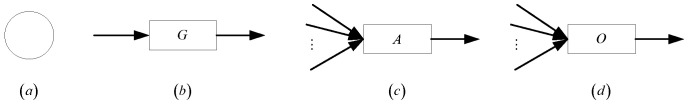
Four types of neurons: (**a**) proposition neuron, (**b**) *G-rule* neuron, (**c**) *A-rule* neuron and (**d**) *O-rule* neuron.

The FRNSN P system is used to model the above three types of fuzzy production rules. Four types of, i.e., proposition, *G-rule*, *A-rule* and *O-rule*, neurons, as shown in Figure 6, are used in the FRNSN P systems. A proposition neuron represents a fuzzy proposition. The *G-rule*, *A-rule* and *O-rule* neurons represent the three types of rules, as discussed below.

**Figure 7:**
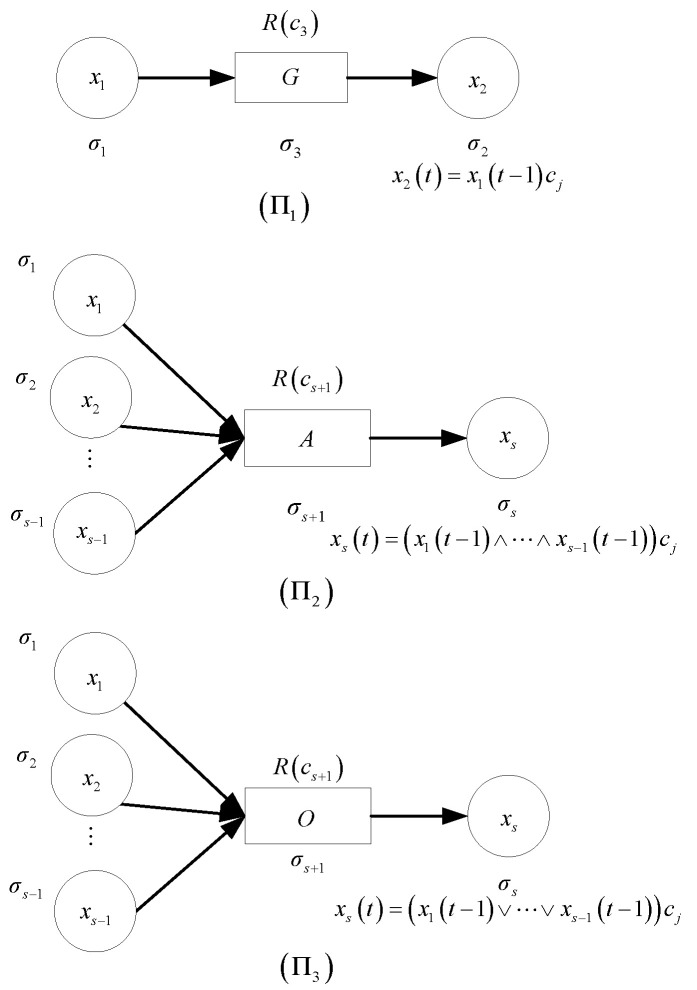
FRNSN P systems modeling the three types of fuzzy production rules.

The *General rule* is modeled by FRNSN P system $\Pi_{1}$ shown in Figure 7 $\left(\Pi_{1}\right)$. System $\Pi_{1}$ is specified in (5) as follows

(5) $$\Pi_{1}=\left(\left\{\sigma_{1},\sigma_{2},\sigma_{3}\right\},syn,in,out\right)$$

The details of the notations used in system $\Pi_{1}$ are given below.

(1) $\sigma_{i}=\left(\theta_{i},Var_{i},{\mathrm{Pr}}_{i},Var_{i}\left(0\right)\right)$ is a proposition neuron representing fuzzy propositions $p_{i}$ for i=1,2;
(2) $\sigma_{3}=\left(\theta_{3},c_{3},Var_{3},{\mathrm{Pr}}_{3},Var_{3}\left(0\right)\right)$ is a *G-rule* neuron;
(3) $syn=\left\{\left(\sigma_{1},\sigma_{3}\right),\left(\sigma_{3},\sigma_{2}\right)\right\}$ is the set of synapses;
(4) $in=\left\{\sigma_{1}\right\}$ and $out=\left\{\sigma_{2}\right\}$ are the input and output proposition neurons.

The fuzzy reasoning process is automatically performed as follows. Initially, the variable of neuron $\sigma_{1}$ is assigned a value of $x_{1}\left(0\right)$. Neuron $\sigma_{1}$ fires and the variable of neuron $\sigma_{3}$ receives the production value $pr_{1}\left(0\right)$ if $pr_{1}\left(0\right)=x_{1}\left(0\right)\geq \theta_{1}$, or does not fire and the value $pr_{1}\left(0\right)$ disappears otherwise, at time t=0. When rule neuron $\sigma_{3}$ satisfies the firing condition, it fires and transmits the production value of $pr_{3}(1)=x_{3}\left(1\right)c_{3}=x_{1}\left(0\right)c_{3}$ to variable $x_{2}$ at time t=1. Thus, the value $x_{1}\left(0\right)c_{3}$ is the result of the computation of system $\Pi_{1}$.

The *AND rule* is modeled by FRNSN P system $\Pi_{2}$ shown in Figure 7 $\left(\Pi_{2}\right)$. System $\Pi_{2}$ is specified in (6) as follows

(6) $$\Pi_{2}=\left(\left\{\sigma_{1},\ldots ,\sigma_{s},\sigma_{s+1}\right\},syn,in,out\right)$$

The details of the notations used in system $\Pi_{2}$ are given below.

(1) $\sigma_{i}=\left(\theta_{i},Var_{i},{\mathrm{Pr}}_{i},Var_{i}\left(0\right)\right)$ is a proposition neuron representing fuzzy proposition $p_{i}$ for 1≤i≤s;
(2) $\sigma_{s+1}=\left(\theta_{s+1},c_{s+1},Var_{s+1},{\mathrm{Pr}}_{s+1},Var_{s+1}\left(0\right)\right)$ is an *A-rule* neuron;
(3) $syn=\left\{\left(\sigma_{1},\sigma_{s+1}\right),\ldots ,\left(\sigma_{s-1},\sigma_{s+1}\right),\left(\sigma_{s+1},\sigma_{s}\right)\right\}$ is the set of synapses;
(4) $in=\left\{\sigma_{1},\ldots ,\sigma_{s-1}\right\}$ and $out=\left\{\sigma_{s}\right\}$ are the set of input neurons and the output neuron.

The fuzzy reasoning process is automatically performed as follows. The variables of neurons $\sigma_{1},\ldots ,\sigma_{s-1}$ are assigned initial values of $x_{1}\left(0\right),\ldots ,x_{s-1}\left(0\right)$, respectively. For 1≤i≤s−1, neuron $\sigma_{i}$ fires and $x_{s+1}\left(t\right)=x_{1}\left(0\right)\wedge \ldots \wedge x_{s-1}\left(0\right)$ if $pr_{i}\left(t\right)\geq \theta_{i}$, and does not fire and $pr_{i}\left(t\right)$ disappears if $pr_{i}\left(t\right)<\theta_{i}$. When the *A-rule* neuron fires the next time, the production value $pr_{s+1}(t)=x_{s+1}\left(t\right)c_{s+1}$ will be transmitted to variable $x_{s}$. Therefore, the result computed by $\Pi_{2}$ is $x_{s}=x_{s+1}\left(t\right)c_{s+1}$.

The *OR rule* is modeled by FRNSN P system $\Pi_{3}$, as shown in Figure 7 $\left(\Pi_{3}\right)$. System $\Pi_{3}$ is specified in (7) as follows

(7) $$\Pi_{3}=\left(\left\{\sigma_{1},\ldots ,\sigma_{s},\sigma_{s+1}\right\},syn,in,out\right)$$

The details of the notations used in system $\Pi_{3}$ are given below:

(1) $\sigma_{i}=\left(\theta_{i},Var_{i},{\mathrm{Pr}}_{i},Var_{i}\left(0\right)\right)$ is a proposition neuron representing fuzzy proposition $p_{i}$ for 1≤i≤s;
(2) $\sigma_{s+1}=\left(\theta_{s+1},c_{s+1},Var_{s+1},{\mathrm{Pr}}_{s+1},Var_{s+1}\left(0\right)\right)$ is the *O-rule* neuron;
(3) $syn=\left\{\left(\sigma_{1},\sigma_{s+1}\right),\ldots ,\left(\sigma_{s-1},\sigma_{s+1}\right),\left(\sigma_{s+1},\sigma_{s}\right)\right\}$ is the set of synapses;
(4) $in=\left\{\sigma_{1},\ldots ,\sigma_{s-1}\right\}$ and $out=\left\{\sigma_{s}\right\}$ are the set of input neurons and the output neuron.

The fuzzy reasoning process of system $\Pi_{3}$ is similar to that of system $\Pi_{2}$, and its description is omitted.

### 4.2. The FRNSN P Reasoning Algorithm

This subsection introduces the FRNSN P reasoning algorithm, as detailed in Algorithm 1. The related matrices, vectors and multiplication operators, as well as a function, are introduced first. The flowchart of the FRNSN P reasoning algorithm is then presented.

(1) $X_{p}\left(t\right)={\left(x_{1}\left(t\right),\ldots ,x_{s}\left(t\right)\right)}^{T}$ is a vector consisting of the fuzzy values of the s variables contained in the s proposition neurons, where $x_{i}\left(t\right)$ is an NIVTFN, for 1≤i≤s;
(2) $X_{r}\left(t\right)={\left(x_{1}\left(t\right),\ldots ,x_{n}\left(t\right)\right)}^{T}$ is a vector consisting of the fuzzy values of the n variables contained in the n rule neurons, where $x_{j}\left(t\right)$ is an NIVTFN, for 1≤j≤n;
(3) $\Theta ={\left(\theta_{1},\ldots ,\theta_{l}\right)}^{T}$ is a vector consisting of the l firing thresholds of the l neurons, where $\theta_{k}$ is an NIVTFN, for 1≤k≤l;
(4) $C=diag\left(c_{1},\ldots c_{n}\right)$ is a diagonal matrix consisting of the confidence factors of the n rule neurons, where $c_{j}$, for 1≤j≤n, is the confidence factor of neuron $\sigma_{j}$, an NIVTFN, representing the credibility of the fuzzy production rule $R_{j}$;
(5) $D_{1}={\left(d_{ij}^{\left(1\right)}\right)}_{s\times n}$ is a matrix representing the synaptic connections from proposition neurons to G−rule neurons, such that $d_{ij}^{\left(1\right)}=1$ if a synapse exists from proposition neuron $\sigma_{i}$ to G−rule neuron $\sigma_{j}$, and $d_{ij}^{\left(1\right)}=0$ otherwise, for 1≤i≤s and 1≤j≤n;
(6) $D_{2}={\left(d_{ij}^{\left(2\right)}\right)}_{s\times n}$ is a matrix representing the synaptic connections from proposition neurons to A−rule neurons, such that $d_{ij}^{\left(2\right)}=1$ if a synapse exists from proposition neuron $\sigma_{i}$ to A−rule neuron $\sigma_{j}$, and $d_{ij}^{\left(2\right)}=0$ otherwise, for 1≤i≤s and 1≤j≤n;
(7) $D_{3}={\left(d_{ij}^{\left(3\right)}\right)}_{s\times n}$ is a matrix representing the synaptic connections from proposition neurons to O−rule neurons such that $d_{ij}^{\left(3\right)}=1$ if a synapse exists from proposition neuron $\sigma_{i}$ to O−rule neuron $\sigma_{j}$, and $d_{ij}^{\left(3\right)}=0$ otherwise, for 1≤i≤s and 1≤j≤n;
(8) $E={\left(e_{ji}\right)}_{n\times s}$ is a matrix representing the synaptic connections from rule neurons to proposition neurons such that $e_{ji}=1$ if a synapse exists from rule neuron $\sigma_{j}$ to proposition neuron $\sigma_{j}$, and $e_{ji}=0$ otherwise, for 1≤i≤s and 1≤j≤n;
(9) $V_{p}\left(t\right)={\left(v_{p1}\left(t\right),\ldots ,v_{ps}\left(t\right)\right)}^{T}$ is a vector consisting of the values passed by proposition neuron $\sigma_{i}$ to the postsynaptic rule neuron variable. If neuron $\sigma_{i}$ does not have a postsynaptic neuron, then this value is passed to the environment as the output value. In particular, $v_{pi}\left(0\right)=0$, for 1≤i≤s;
(10) $V_{r}\left(t\right)={\left(v_{r1}\left(t\right),\ldots ,v_{rn}\left(t\right)\right)}^{T}$ is a vector consisting of the values passed by rule neuron $\sigma_{j}$ to the postsynaptic proposition neuron variable. In particular, $v_{rj}\left(0\right)=0$ for 1≤j≤n.

In addition, several multiplication operators for the above matrices and vectors are defined:

(1) $C⊗X_{r}\left(t\right)={\left(c_{1}x_{1}\left(t\right),\ldots ,c_{n}x_{n}\left(t\right)\right)}^{T}$. Similarly, $D_{1}{}^{T}⊗X_{p}\left(t\right)={\left({\bar{d}}_{1}^{\left(1\right)},{\bar{d}}_{2}^{\left(1\right)},\ldots {\bar{d}}_{n}^{\left(1\right)}\right)}^{T}$, where ${\bar{d}}_{j}^{\left(1\right)}=d_{1j}^{\left(1\right)}x_{1}\left(t\right)+d_{2j}^{\left(1\right)}x_{2}\left(t\right)+\ldots +d_{sj}^{\left(1\right)}x_{s}\left(t\right)$, for 1≤j≤n;
(2) $D_{2}^{T}⊙X_{p}\left(t\right)={\left({\bar{d}}_{1}^{\left(2\right)},{\bar{d}}_{2}^{\left(2\right)},\ldots {\bar{d}}_{n}^{\left(2\right)}\right)}^{T}$, where ${\bar{d}}_{j}^{\left(2\right)}=d_{1j}^{\left(2\right)}x_{1}\left(t\right)\vee d_{2j}^{\left(2\right)}x_{2}\left(t\right)\vee \ldots \vee d_{sj}^{\left(2\right)}x_{s}\left(t\right)$, for 1≤j≤n;
(3) $D_{3}^{T}⊕X_{p}\left(t\right)={\left({\bar{d}}_{1}^{\left(3\right)},{\bar{d}}_{2}^{\left(3\right)},\ldots {\bar{d}}_{n}^{\left(3\right)}\right)}^{T}$, where ${\bar{d}}_{j}^{\left(3\right)}=d_{1j}^{\left(3\right)}x_{1}\left(t\right)\wedge d_{2j}^{\left(3\right)}x_{2}\left(t\right)\wedge \ldots \wedge d_{sj}^{\left(3\right)}x_{s}\left(t\right)$, for 1≤j≤n.

Finally, a function (8) for production value $pr_{k}\left(t\right)$ and threshold θ is defined.

(8) $$v_{k}\left(t\right)=\{\begin{array}{ll} pr_{k}\left(t\right), & \mathrm{if}\;pr_{k}\left(t\right)\geq \theta_{k}\;\\ 0, & \mathrm{otherwise} \end{array},\;1\leq k\leq l,\;s+n=l.$$

**Algorithm 1**: The FRNSN P reasoning algorithm**Input**: Θ, C, $D_{1}$, $D_{2}$, $D_{3}$, E, $X_{P}\left(0\right)$, $X_{r}\left(0\right)$
Let t=0;
Set the stopping condition $0_{r}={\left(0,\ldots ,0\right)}_{n}^{T}$;   **while** ($X_{r}\left(t\right)\neq 0_{r}$) **do**
     **for** each of the (input) proposition neurons **do**
         **if** the proposition neuron has a postsynaptic rule neuron **then**
             Calculate $X_{r}\left(t\right)=\left(D_{1}^{T}⊗V_{p}\left(t\right)\right)+\left(D_{2}^{T}⊙V_{p}\left(t\right)\right)+\left(D_{3}^{T}⊕V_{p}\left(t\right)\right)$;              **if** $pr_{i}\left(t\right)\geq \theta_{i}$ **then**
                Transmits the value $v_{pi}\left(t\right)$ to the rule neuron;              **else**
                Transmits the value 0 to the rule neuron;              **end if**         **end if**
     **end for**
     **for** each of the rule neurons **do**
         **if** $pr_{j}\left(t\right)\geq \theta_{j}$ **then**
             Transmits the value $v_{rj}\left(t\right)$ to the postsynaptic proposition neuron;              Calculate $X_{p}\left(t\right)=E^{T}⊕\left(C⊗V_{r}\left(t\right)\right)$;          **end if**       **end for**
     t=t+1; 
**end while**

**Output**: The fuzzy values of the output proposition neurons.

**Figure 8:**
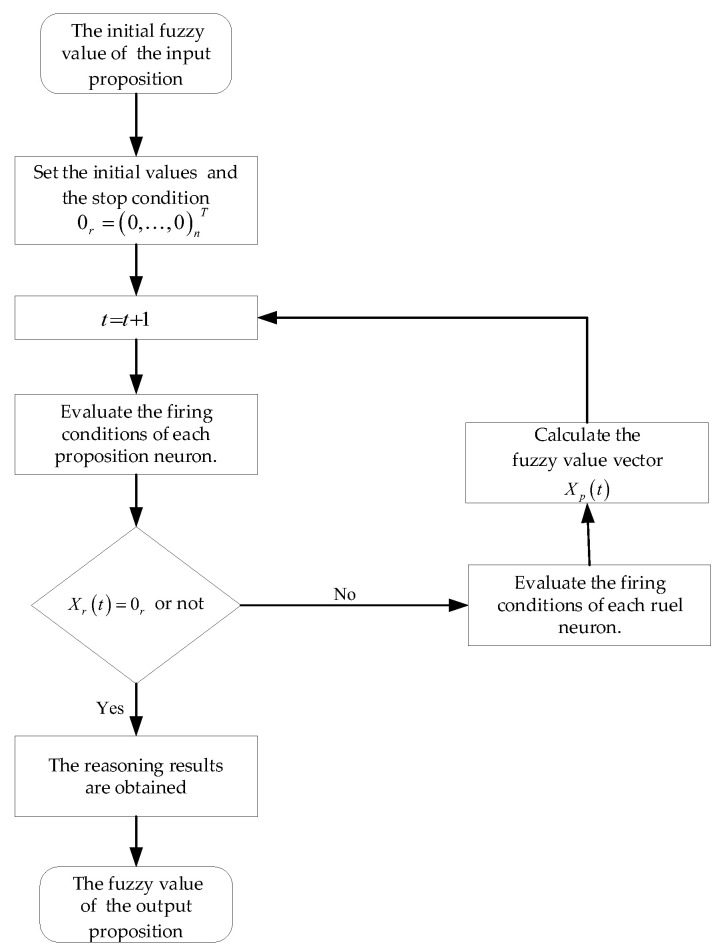
The flowchart of the FRNSN P reasoning algorithm.

Matrices Θ, C and $X_{p}\left(0\right)$ were obtained from expert experience and historical data, and matrices $D_{1}$, $D_{2}$, *D*_3_ and *E* were obtained from the topology of the FRNSN P system. The flowchart of the FRNSN P reasoning algorithm is shown in Figure 8.

## 5. Fault Diagnosis of Induction Motors Using the FRNSN P Reasoning Algorithm

**Figure 9:**
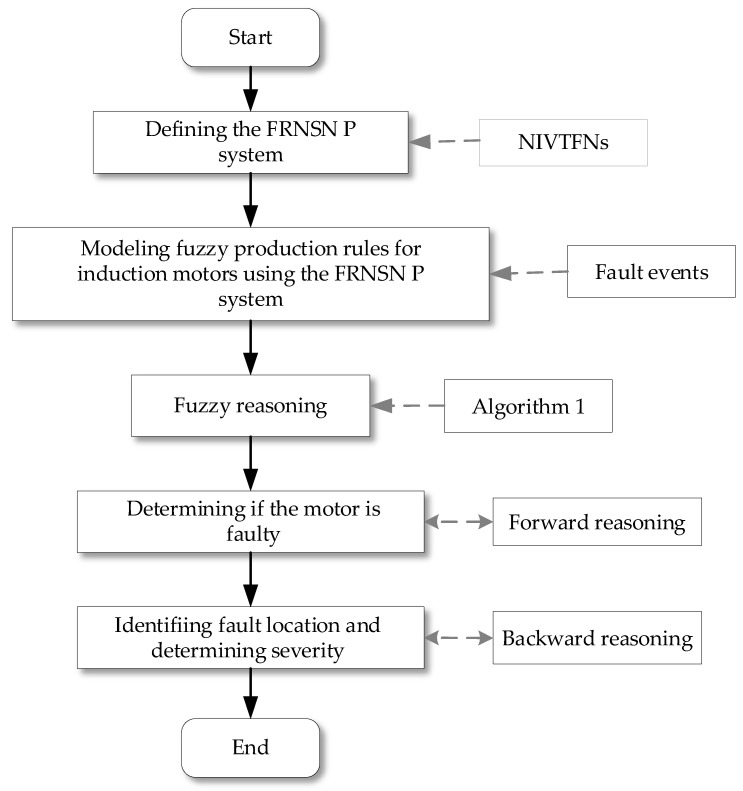
The flowchart of induction motor fault diagnosis process using Algorithm 1.

The fault mechanism of induction motors is complex, and the relationship between a fault and a symptom is not one-to-one correspondent but is complex. Generally, a fault manifests as multiple symptoms and different faults may correspond to the same symptom [37,38,39]. The faults of induction motors are mostly related to windings, bearings and rotors. The single-fault cases “Winding insulation burnt”, “Bearing damage” and “Broken rotor bar”, and the multiple-fault cases “Winding insulation burnt and bearing damage” and “Bearing damage and broken rotor bar”, as listed in Table 3, were investigated using Algorithm 1. Due to the similarity of the reasoning processes, the multi-fault case “Winding insulation burnt and bearing damage” is used as an example for detailed description. A flowchart showing the induction motor fault diagnosis process is in Figure 9.

### 5.1. Fuzzy Production Rules for Induction Motors

**Figure 10:**
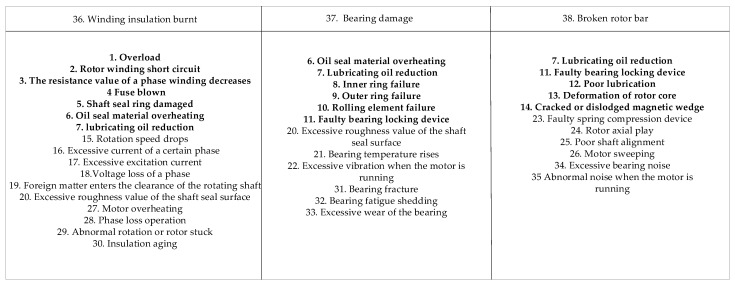
Fault events related to motor faults.

The fuzzy production rules related to motor faults are presented and the relevant fault events are enumerated, as shown in Figure 10 [37,38,40]. There is a one-to-one correspondence between fault events and propositions in fuzzy production rules. Fault events 36, 37 and 38 are the immediate causes of “motor fault”, and the motor is considered faulty whichever of the three faults occurs. The events in bold in Figure 10 are fault symptom events of faults 36, 37 and 38, and event 7 is a symptom of all the three faults. The fuzzy production rules are listed below:

$R_{1}$: IF $p_{1}$, THEN $P_{15}$ $\left(C=c_{1}\right)$;

$R_{2}$: IF $p_{2}$ AND $p_{3}$, THEN $P_{16}$ $\left(C=c_{2}\right)$;

$R_{3}$: IF $p_{3}$, THEN $P_{17}$ $\left(C=c_{3}\right)$;

$R_{4}$: IF $p_{4}$, THEN $P_{18}$ $\left(C=c_{4}\right)$;

$R_{5}$: IF $p_{5}$, THEN $P_{19}$ $\left(C=c_{5}\right)$;

$R_{6}$: IF $p_{6}$ OR $p_{7}$, THEN $P_{20}$ $\left(C=c_{6}\right)$;

$R_{7}$: IF $p_{8}$ OR $p_{9}$, THEN $P_{21}$ $\left(C=c_{7}\right)$;

$R_{8}$: IF $p_{10}$ OR $p_{11}$, THEN $P_{22}$ $\left(C=c_{8}\right)$;

$R_{9}$: IF $p_{11}$, THEN $P_{23}$ $\left(C=c_{9}\right)$;

$R_{10}$: IF $p_{12}$, THEN $P_{24}$ $\left(C=c_{10}\right)$;

$R_{11}$: IF $p_{7}$, THEN $P_{25}$ $\left(C=c_{11}\right)$;

$R_{12}$: IF $p_{13}$ OR $p_{14}$, THEN $P_{26}$ $\left(C=c_{12}\right)$;

$R_{13}$: IF $p_{15}$ OR $p_{16}$ OR $p_{17}$, THEN $P_{27}$ $\left(C=c_{13}\right)$;

$R_{14}$: IF $p_{18}$, THEN $P_{28}$ $\left(C=c_{14}\right)$;

$R_{15}$: IF $p_{19}$, THEN $P_{29}$ $\left(C=c_{15}\right)$;

$R_{16}$: IF $p_{20}$, THEN $P_{30}$ $\left(C=c_{16}\right)$;

$R_{17}$: IF $p_{20}$, THEN $P_{31}$ $\left(C=c_{17}\right)$;

$R_{18}$: IF $p_{21}$, THEN $P_{32}$ $\left(C=c_{18}\right)$;

$R_{19}$: IF $p_{22}$, THEN $P_{33}$ $\left(C=c_{19}\right)$;

$R_{20}$: IF $p_{23}$ OR $p_{24}$ OR $p_{25}$, THEN $P_{34}$ $\left(C=c_{20}\right)$;

$R_{21}$: IF $p_{26}$, THEN $P_{35}$ $\left(C=c_{21}\right)$;

$R_{22}$: IF $p_{27}$ OR $p_{28}$ OR $p_{29}$ OR $p_{30}$, THEN $P_{36}$ $\left(C=c_{22}\right)$;

$R_{23}$: IF $p_{31}$ OR $p_{32}$ OR $p_{33}$, THEN $P_{37}$ $\left(C=c_{23}\right)$;

$R_{24}$: IF $p_{34}$ OR $p_{35}$, THEN $P_{38}$ $\left(C=c_{24}\right)$;

$R_{25}$: IF $p_{36}$ OR $p_{37}$ OR $p_{38}$, THEN $P_{39}$ $\left(C=c_{25}\right)$.

### 5.2. Parameter Settings

The relevant parameters of the FRNSN P reasoning algorithm are specified in this subsection. The confidence factors $c_{j}$ for 1≤j≤n of the *O-rule* neurons, the *G-rule* neurons and the *A-rule* neurons were set to EH=[(1.00, 1.00), 1.00, (1.00, 1.00)], VH=[(0.86,0.90),0.93,(0.97,1)] and H=[(0.73,0.79),0.82,(0.84,0.90)], respectively, based on experience and historical data [39,40]. The firing thresholds $\theta_{k}$ for 1≤k≤l of the proposition neurons and the rule neurons were set to M=[(0.454,0.48),0.52,(0.55,0.64)]. In addition, if the NIVTFN of the variable in a proposition neuron satisfies $x_{i}\left(t\right)\geq FH=\left[\left(0.62,0.642\right),0.67,\left(0.721,0.78\right)\right]$, then the fault event corresponding to the proposition neuron has indeed occurred. If the NIVTFN of the variable in a proposition neuron satisfies $x_{i}\left(t\right)\leq FL=\left[\left(0.33,0.36\right),0.44,\left(0.46,0.52\right)\right]$, then the fault event corresponding to the proposition neuron has not occurred.

### 5.3. Case Studies

**Figure 11:**
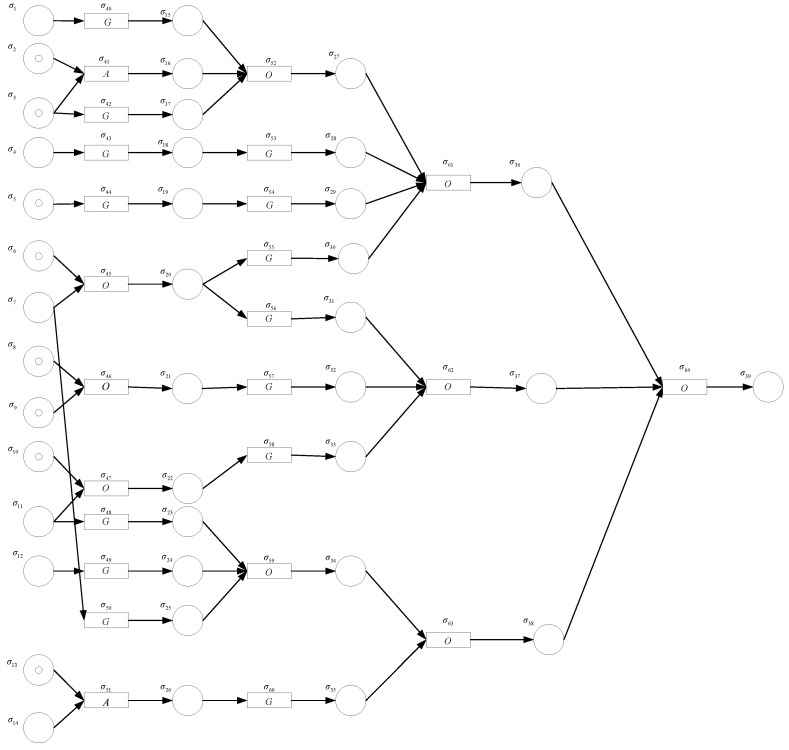
The forward reasoning model for induction motor fault diagnosis using the FRNSN P system.

In this subsection, the potential fault of the motor is modeled using the fuzzy production rules, as shown in Figure 11. The fault diagnosis of the motor was carried out through Algorithm 1. Specifically, fault diagnosis contains two phases. The first phase is forward reasoning, which is to infer whether the motor will fail according to the probability of occurrence of failure events. The second phase is backward reasoning, that is to infer the fault cause and fault path of the motor after determining the motor fault. Suppose that the fault symptom events 2, 3, 5, 6, 8, 9, 10 and 13 occurred according to the online monitoring system, indicating that the initial NIVTFNs of the variables in neurons $\sigma_{2}$, $\sigma_{3}$, $\sigma_{5}$, $\sigma_{6}$, $\sigma_{8}$, $\sigma_{9}$, $\sigma_{10}$ and $\sigma_{13}$ are all greater than or equal to FH as defined in Table 2.

#### 5.3.1. Forward Reasoning

The threshold vector Θ and the confidence factor matrix C were presented in Section 5.2. The synaptic connection matrices $D_{1}$, $D_{2}$, $D_{3}$ and E are given in the topological structure of the FRNSN P system in Figure 11. The initial IVTFNs of the variables of the input proposition neurons, i.e., the probabilities of occurrences of fault symptom events, were obtained according to the historical data and the experienced fault diagnosis reports in the industry [39,40].

The detailed process of forward reasoning using the FRNSN P system in Figure 11 is as follows. Initially only the variables of the input proposition neurons contain nonzero values. A **0** represents a vector of 0 s, i.e., the NIVTFNs in the neurons are [(0,0),0,(0,0)]. An input proposition neuron fires and passes the production value to the rule neurons if it satisfies the threshold condition and does not fire and the contained production value disappears otherwise. The neurons in Figure 11 fire hierarchically and the production values are passed from presynaptic to postsynaptic neurons. According to the fuzzy reasoning process of the three FRNSN P systems in Section 4.1, the values of the variables, represented by the NIVTFNs, in the neurons at each time step are as follows.

$X_{p}\left(0\right)=\left[\begin{array}{c} \left[\left(0.33,0.36\right),0.44,\left(0.46,0.52\right)\right]\\ \left[\left(0.62,0.642\right),0.67,\left(0.721,0.78\right)\right]\\ \left[\left(0.86,0.90\right),0.93,\left(0.97,1\right)\right]\\ \left[\left(0.20,0.24\right),0.27,\left(0.30,0.39\right)\right]\\ \left[\left(0.86,0.90\right),0.93,\left(0.97,1\right)\right]\\ \left[\left(0.73,0.79\right),0.82,\left(0.84,0.90\right)\right]\\ \left[\left(0.20,0.24\right),0.27,\left(0.30,0.39\right)\right]\\ \left[\left(0.73,0.79\right),0.82,\left(0.84,0.90\right)\right]\\ \left[\left(0.73,0.79\right),0.82,\left(0.84,0.90\right)\right]\\ \left[\left(0.62,0.642\right),0.67,\left(0.721,0.78\right)\right]\\ \left[\left(0.20,0.24\right),0.27,\left(0.30,0.39\right)\right]\\ \left[\left(0,0.06\right),0.12,\left(0.18,0.23\right)\right]\\ \left[\left(0.62,0.642\right),0.67,\left(0.721,0.78\right)\right]\\ \left[\left(0.20,0.24\right),0.27,\left(0.30,0.39\right)\right]\\ 0 \end{array}\right]$, $X_{r}\left(0\right)=\left[0\right]$.

When t=1,

$X_{r}\left(1\right)=\left[\begin{array}{c} \left[\left(0,0\right),0,\left(0,0\right)\right]\\ \left[\left(0.62,0.642\right),0.67,\left(0.721,0.78\right)\right]\\ \left[\left(0.86,0.90\right),0.93,\left(0.97,1\right)\right]\\ \left[\left(0,0\right),0,\left(0,0\right)\right]\\ \left[\left(0.86,0.90\right),0.93,\left(0.97,1\right)\right]\\ \left[\left(0.73,0.79\right),0.82,\left(0.84,0.90\right)\right]\\ \left[\left(0.73,0.79\right),0.82,\left(0.84,0.90\right)\right]\\ \left[\left(0.62,0.642\right),0.67,\left(0.721,0.78\right)\right]\\ 0 \end{array}\right]$, $X_{p}\left(1\right)=\left[\begin{array}{c} 0\\ \left[\left(0.7396,0.81\right),0.8649,\left(0.9409,1\right)\right]\\ \left[\left(0,0\right),0,\left(0,0\right)\right]\\ \left[\left(0.7396,0.81\right),0.8649,\left(0.9409,1\right)\right]\\ \left[\left(0.73,0.79\right),0.82,\left(0.84,0.9\right)\right]\\ \left[\left(0.73,0.79\right),0.82,\left(0.84,0.9\right)\right]\\ \left[\left(0.62,0.642\right),0.67,\left(0.721,0.78\right)\right]\\ 0 \end{array}\right]$.

When t=2,

$X_{r}\left(2\right)=\left[\begin{array}{c} 0\\ \left[\left(0.7396,0.81\right),0.8649,\left(0.9409,1\right)\right]\\ \left[\left(0,0\right),0,\left(0,0\right)\right]\\ \left[\left(0.7396,0.81\right),0.8649,\left(0.9409,1\right)\right]\\ \left[\left(0.73,0.79\right),0.82,\left(0.84,0.9\right)\right]\\ \left[\left(0.73,0.79\right),0.82,\left(0.84,0.9\right)\right]\\ \left[\left(0.73,0.79\right),0.82,\left(0.84,0.9\right)\right]\\ \left[\left(0.62,0.642\right),0.67,\left(0.721,0.78\right)\right]\\ 0 \end{array}\right]$, $X_{p}\left(2\right)=\left[\begin{array}{c} 0\\ \left[\left(0.7396,0.81\right),0.8649,\left(0.9409,1\right)\right]\\ \left[\left(0,0\right),0,\left(0,0\right)\right]\\ \left[\left(0.6361,0.729\right),0.8044,\left(0.9127,1\right)\right]\\ \left[\left(0.6278,0.711\right),0.7626,\left(0.8148,0.9\right)\right]\\ \left[\left(0.6278,0.711\right),0.7626,\left(0.8148,0.9\right)\right]\\ \left[\left(0.6278,0.711\right),0.7626,\left(0.8148,0.9\right)\right]\\ \left[\left(0.5332,0.5778\right),0.6231,\left(0.6994,0.78\right)\right]\\ 0 \end{array}\right]$.

When t=3,

$X_{r}\left(3\right)=\left[\begin{array}{c} 0\\ \left[\left(0.7396,0.81\right),0.8649,\left(0.9409,1\right)\right]\\ \left[\left(0.6278,0.711\right),0.7626,\left(0.8148,0.9\right)\right]\\ 0 \end{array}\right]$, $X_{p}\left(3\right)=\left[\begin{array}{c} 0\\ \left[\left(0.7396,0.81\right),0.8649,\left(0.9409,1\right)\right]\\ \left[\left(0.6278,0.711\right),0.7626,\left(0.8148,0.9\right)\right]\\ 0 \end{array}\right]$.

When t=4,

$X_{r}\left(4\right)=\left[\begin{array}{c} 0\\ \left[\left(0.7396,0.81\right),0.8649,\left(0.9409,1\right)\right]\\ 0 \end{array}\right]$, $X_{p}\left(4\right)=\left[\begin{array}{c} 0\\ \left[\left(0.7396,0.81\right),0.8649,\left(0.9409,1\right)\right]\\ 0 \end{array}\right]$.

When t=5,

$$X_{r}\left(5\right)=\left[0\right]$$

When the computation completes at t=4, the value of the variable in the output proposition neuron $\sigma_{39}$ is [(0.7396,0.81),0.8649,(0.9409,1)]. The output proposition neuron $\sigma_{39}$ fires since it satisfies the firing condition [(0.7396,0.81),0.8649,(0.9409,1)]≥M at t=5. Therefore, $X_{r}\left(5\right)=\left[0\right]$, the stopping condition is satisfied, the algorithm terminates and the reasoning result is obtained. The fault event corresponding to the output proposition neuron $\sigma_{39}$ occurs, i.e., the motor is faulty, since [(0.7396,0.81),0.8649,(0.9409,1)]≥FH.

#### 5.3.2. Backward Reasoning

**Figure 12:**
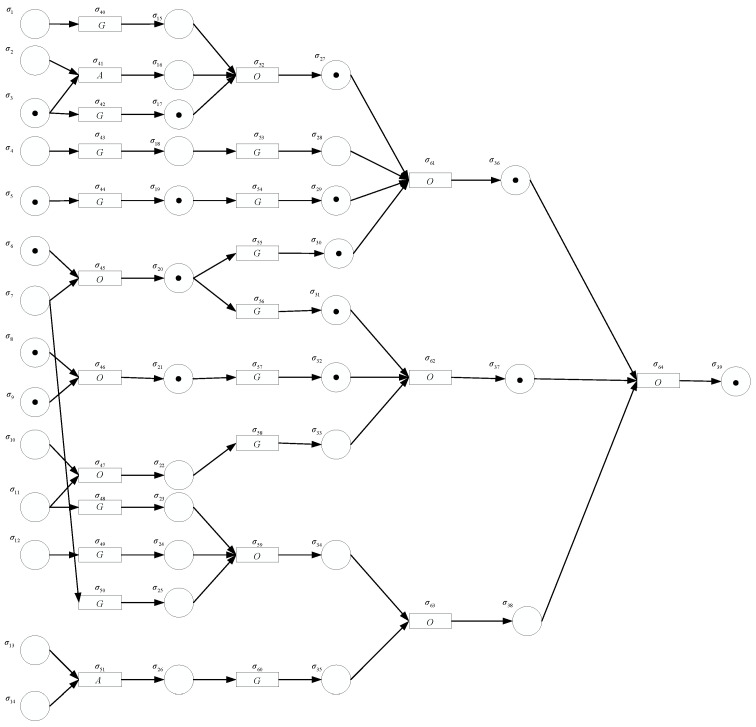
The backward reasoning model for induction motor fault diagnosis using the FRNSN P system.

After the induction motor is determined to be faulty, the computation results of the FRNSN P reasoning algorithm are used to perform backward reasoning to find out the fault event, fault source and the fault propagation path. The backward reasoning model is shown in Figure 12.

The immediate cause of the motor failure can be determined from the threshold conditions. Since the confidence factors of propositions $p_{36}$, $p_{37}$ and $p_{38}$ are [(0.7396,0.81),0.8649,(0.9409,1)]≥FH, [(0.6278,0.711),0.7626,(0.8148,0.90)]≥FH and [(0.2838,0.324),0.4092,(0.4462,0.52)]≤FL, respectively, the fault events “Winding insulation burnt” and “Bearing damage”, but not “Broken rotor bar”, are determined to have occurred.

The fault propagation path generally begins with the source of the fault and ends at the immediate cause of the motor fault. In this case, there are six fault propagation paths, including $L_{1}=\left\{\sigma_{3}\to \sigma_{17}\to \sigma_{27}\to \sigma_{36}\to \sigma_{39}\right\}$, $L_{2}=\left\{\sigma_{5}\to \sigma_{19}\to \sigma_{29}\to \sigma_{36}\to \sigma_{39}\right\}$, $L_{3}=\left\{\sigma_{6}\to \sigma_{20}\to \sigma_{30}\to \sigma_{36}\to \sigma_{39}\right\}$, $L_{4}=\left\{\sigma_{6}\to \sigma_{20}\to \sigma_{31}\to \sigma_{37}\to \sigma_{39}\right\}$, $L_{5}=\left\{\sigma_{8}\to \sigma_{21}\to \sigma_{32}\to \sigma_{37}\to \sigma_{39}\right\}$ and $L_{6}=\left\{\sigma_{9}\to \sigma_{21}\to \sigma_{32}\to \sigma_{37}\to \sigma_{39}\right\}$. It can be found that the fault events 2, 10 and 13 cannot ultimately lead to motor failure even if they also occur, i.e., they are not fault sources for motor failure.

Next, the severity of “Winding insulation burnt” and “Bearing failure” are determined by computing the relative preference values for proposition neurons $\sigma_{37}$ and $\sigma_{38}$. Let $A_{1}=\left[\left(0.7396,0.81\right),0.8649,\left(0.9409,1\right)\right]$ and $A_{2}=\left[\left(0.6278,0.711\right),0.7626,\left(0.8148,0.90\right)\right]$, then $\bar{A}=\left[\left(0.6837,0.7605\right),0.81375,\left(0.87785,0.95\right)\right]$ $T_{S}^{L-}=\left(t_{sl}^{L-},t_{sh}^{-},t_{sr}^{L-}\right)=\left(0.711,0.7626,08148\right)$, $T_{S}^{L+}=\left(t_{sl}^{L+},t_{sh}^{+},t_{sr}^{L+}\right)=\left(0.81,08649,0.9409\right)$, $T_{S}^{U-}=\left(t_{sl}^{U-},t_{sh}^{-},t_{sr}^{U-}\right)=\left(0.6278,0.7626,0.9\right)$, $T_{S}^{U+}=\left(t_{sl}^{U+},t_{sh}^{+},t_{sr}^{U+}\right)=\left(0.7396,0.8649,1\right)$, $\left\|T_{S}^{L+},T_{S}^{L-}\right\|=0.22445$, $\left\|T_{S}^{U+},T_{S}^{U-}\right\|=0.529$, $\mu_{\beta }\left(A_{1},\bar{A}\right)=0.669$ and $\mu_{\beta }\left(A_{2},\bar{A}\right)=0.331$. Due to the relative preference value $\mu_{\beta }\left(A_{1},\bar{A}\right)>\mu_{\beta }\left(A_{2},\bar{A}\right)$, the fault of “Winding insulation burnt” is more serious. When multiple faults occur in the motor, the introduction of the relative preference relation β can help determine the severities of all the faults.

Finally, the performance of the FRNSN P system was compared with those of other motor fault diagnosis methods. The methods used for comparison were FFPN [37], CLPSO-FPN [38] and rMFRSNPs [40], and the comparison results are shown in Table 3. Fault events, fault symptoms, fault sources and fault cases are represented by corresponding neurons.

For the single-fault cases “Winding insulation burnt” and “Broken rotor bar”, FFPN [37], CLPSO-FPN [38], rMFRSNPs [40] and FRNSN P could correctly detect the fault events and obtain the same fault sources and fault causes. The fault event of case 3 was “Bearing damage”. Although all four methods could obtain the correct detection results, FFPN [37] and CLPSO-FPN [38] found one more fault source, i.e., neuron $\sigma_{9}$, than rMFRSNPs [40] and FRNSN P did.

For the multi-fault case “Bearing damage and broken rotor bar”, CLPSO-FPN [38], rMFRSNPs [40] and FRNSN P gave correct and consistent results, but FFPN [37] could only detect one of the faults, i.e., “Broken rotor bar”. For the multi-fault case “Winding insulation burnt and bearing damage” detailed in this subsection, FRNSN P showed certain advantages, i.e., it could correctly detect faults “Winding insulation burnt” and “Bearing damage”, but FFPN [37] and CLPSO-FPN [38] could only detect fault “Winding insulation burnt”. Although rMFRSNPs [40] could also detect faults “Winding insulation burnt” and “Bearing damage”, fault “Broken rotor bar” that did not exist was also detected. In addition, the fault sources were slightly different for each method. Since FFPN and CLPSO-FPN could only detect fault event 36, their fault sources were only associated with event 36. Since rMFRSNPs detected one more fault, it found more fault sources than FRNSN P did. For the same case, it is reasonable and acceptable for different methods to have slightly different fault sources due to different operating mechanisms and parameter settings.

## 6. Conclusions

In this work, the NSN P systems were extended to the FRNSN P systems by introducing IVTFNs. FRNSN P systems can easily model the fuzzy production rules of motor faults. A fuzzy reasoning algorithm based on the FRNSN P system was proposed for motor fault diagnosis. Through the study of single fault and multiple fault cases, the effectiveness and feasibility of the FRNSN P reasoning algorithm were proved for motor fault diagnosis. In addition, the relative preference relationship can be used to estimate the severity of various faults, so that the motor can be repaired in time when a minor fault occurs to prevent the fault from worsening.

Since it is necessary to rely on historical data and expert experience to obtain the probability of occurrence of motor fault symptoms, signal processing technology will be combined with the FRNSN P system to obtain real-time motor fault information in a future study. Specifically, considering that the stator current signal is minimally affected by the external environment and the current sensor is easy to install, the current signal will be used to obtain fault information. According to the fault information, the occurrence probability of some cause events can be obtained early, the fault probability corresponding to the IVTFN can then be estimated, and the FRNSN P reasoning algorithm is finally used for fault diagnosis. Furthermore, other intelligent algorithms can be introduced into the FRNSN P system so as to apply it to other real-world applications including the fault diagnosis of other types of motors.

## Figures and Tables

**Table 1:** Comparisons of time steps of different P systems for solving the SAT problem.

Modules	Time Steps
NSN P systems	6
DDSN P systems [34]	2n+m+3
WSN P systems [35]	2n+m+3
SN P systems with neuron division and budding [36]	2n+mn+6

**Table 2:** The correspondence between linguistic terms and NIVTFNs.

Linguistic Terms	NIVTFNs
Extremely Low (EL)	[(0,0),0,(0,0)]
Very Low (VL)	[(0,0.06),0.12,(0.18,0.23)]
Low (L)	[(0.20,0.24),0.27,(0.30,0.39)]
Fairly Low (FL)	[(0.33,0.36),0.44,(0.46,0.52)]
Medium (M)	[(0.454,0.48),0.52,(0.55,0.64)]
Fairly High (FH)	[(0.62,0.642),0.67,(0.721,0.78)]
High (H)	[(0.73,0.79),0.82,(0.84,0.90)]
Very High (VH)	[(0.86,0.90),0.93,(0.97,1)]
Extremely High (EH)	[(1.00, 1.00), 1.00, (1.00, 1.00)]

**Table 3:** Comparisons of the reasoning results of FRNSN P and the other three methods.

	Preset				Methods	Result		
	Cases	Fault Locations	Fault Symptoms	Fault Cases		Fault Events	Fault Sources	Fault Cases
1	Broken rotor bar	$\sigma_{38}$	$\sigma_{12}(H)$	$\sigma_{12},\sigma_{23},\sigma_{34}$	FFPN [37]	$\sigma_{38}$	$\sigma_{12}$	$\sigma_{12},\sigma_{23},\sigma_{34}$
					CLPSO-FPN [38]	$\sigma_{38}$	$\sigma_{12}$	$\sigma_{12},\sigma_{23},\sigma_{34}$
					rMFRSNPs [40]	$\sigma_{38}$	$\sigma_{12}$	$\sigma_{12},\sigma_{23},\sigma_{34}$
					FRNSN P	$\sigma_{38}$	$\sigma_{12}$	$\sigma_{12},\sigma_{23},\sigma_{34}$
2	Winding insulation burnt	$\sigma_{36}$	$\sigma_{2}(FH),\sigma_{3}(H)$	$\begin{array}{c} \sigma_{2},\sigma_{3},\sigma_{17},\\ \sigma_{27} \end{array}$	FFPN [37]	$\sigma_{36}$	$\sigma_{2},\sigma_{3}$	$\begin{array}{c} \sigma_{2},\sigma_{3},\sigma_{17},\\ \sigma_{27} \end{array}$
					CLPSO-FPN [38]	$\sigma_{36}$	$\sigma_{2},\sigma_{3}$	$\begin{array}{c} \sigma_{2},\sigma_{3},\sigma_{17},\\ \sigma_{27} \end{array}$
					rMFRSNPs [40]	$\sigma_{36}$	$\sigma_{2},\sigma_{3}$	$\begin{array}{c} \sigma_{2},\sigma_{3},\sigma_{17},\\ \sigma_{27} \end{array}$
					FRNSN P	$\sigma_{36}$	$\sigma_{2},\sigma_{3}$	$\begin{array}{c} \sigma_{2},\sigma_{3},\sigma_{17},\\ \sigma_{27} \end{array}$
3	Bearing damage	$\sigma_{37}$	$\sigma_{8}(H),\sigma_{9}(FH)$	$\sigma_{8},\sigma_{21},\sigma_{32}$	FFPN [37]	$\sigma_{37}$	$\sigma_{8},\sigma_{9}$	$\begin{array}{c} \sigma_{8},\sigma_{9},\sigma_{21},\\ \sigma_{32} \end{array}$
					CLPSO-FPN [38]	$\sigma_{37}$	$\sigma_{8},\sigma_{9}$	$\begin{array}{c} \sigma_{8},\sigma_{9},\sigma_{21},\\ \sigma_{32} \end{array}$
					rMFRSNPs [40]	$\sigma_{37}$	$\sigma_{8}$	$\sigma_{8},\sigma_{21},\sigma_{32}$
					FRNSN P	$\sigma_{37}$	$\sigma_{8}$	$\sigma_{8},\sigma_{21},\sigma_{32}$
4	Bearing damage and broken rotor bar	$\sigma_{37},\sigma_{38}$	$\begin{array}{c} \sigma_{8}(H),\sigma_{10}(FH),\\ \sigma_{11}(VH) \end{array}$	$\begin{array}{c} \sigma_{8},\sigma_{11},\\ \sigma_{21},\sigma_{22},\sigma_{23},\\ \sigma_{32},\sigma_{33},\sigma_{34} \end{array}$	FFPN [37]	$\sigma_{38}$	$\sigma_{11}$	$\sigma_{11},\sigma_{23},\sigma_{34}$
					CLPSO-FPN [38]	$\sigma_{37},\sigma_{38}$	$\sigma_{8},\sigma_{11}$	$\begin{array}{c} \sigma_{8},\sigma_{11},\\ \sigma_{22},\sigma_{23},\\ \sigma_{32},\sigma_{33},\sigma_{34} \end{array}$
					rMFRSNPs [40]	$\sigma_{37},\sigma_{38}$	$\sigma_{8},\sigma_{11}$	$\begin{array}{c} \sigma_{8},\sigma_{11},\\ \sigma_{21},\sigma_{22},\sigma_{23},\\ \sigma_{32},\sigma_{33},\sigma_{34} \end{array}$
					FRNSN P	$\sigma_{37},\sigma_{38}$	$\sigma_{8},\sigma_{11}$	$\begin{array}{c} \sigma_{8},\sigma_{11},\\ \sigma_{21},\sigma_{22},\sigma_{23},\\ \sigma_{32},\sigma_{33},\sigma_{34} \end{array}$
5	Winding insulation burnt and bearing damage	$\sigma_{36},\sigma_{37}$	$\begin{array}{c} \sigma_{2}(FH),\sigma_{3}(VH),\\ \sigma_{5}(VH),\sigma_{6}(H),\\ \sigma_{8}(H),\sigma_{9}(H),\\ \sigma_{10}(FH),\\ \sigma_{13}(FH) \end{array}$	$\begin{array}{c} \sigma_{3},\sigma_{5},\sigma_{6},\\ \sigma_{8},\sigma_{9},\sigma_{17},\\ \sigma_{19},\sigma_{20},\sigma_{21},\\ \sigma_{27},\sigma_{29},\sigma_{30},\\ \sigma_{31},\sigma_{32} \end{array}$	FFPN [37]	$\sigma_{36}$	$\sigma_{3},\sigma_{5},\sigma_{6}$	$\begin{array}{c} \sigma_{3},\sigma_{5},\sigma_{6},\\ \sigma_{17},\sigma_{20},\sigma_{27},\\ \sigma_{30} \end{array}$
					CLPSO-FPN [38]	$\sigma_{36}$	$\sigma_{3},\sigma_{5},\sigma_{6}$	$\begin{array}{c} \sigma_{3},\sigma_{5},\sigma_{6},\\ \sigma_{17},\sigma_{20},\sigma_{27},\\ \sigma_{30} \end{array}$
					rMFRSNPs [40]	$\begin{array}{c} \sigma_{36},\sigma_{37},\\ \sigma_{38} \end{array}$	$\begin{array}{c} \sigma_{2},\sigma_{3},\sigma_{5},\\ \sigma_{6},\sigma_{8},\sigma_{9},\\ \sigma_{13} \end{array}$	$\begin{array}{c} \sigma_{2},\sigma_{3},\sigma_{5},\\ \sigma_{6},\sigma_{8},\sigma_{9},\\ \sigma_{13},\sigma_{17},\sigma_{19}\\ \sigma_{20},\sigma_{21},\sigma_{27}\\ \sigma_{29},\sigma_{30},\\ \sigma_{31},\sigma_{32} \end{array}$
					FRNSN P	$\sigma_{36},\sigma_{37}$	$\begin{array}{c} \sigma_{3},\sigma_{5},\sigma_{6},\\ \sigma_{8},\sigma_{9} \end{array}$	$\begin{array}{c} \sigma_{3},\sigma_{5},\sigma_{6},\\ \sigma_{8},\sigma_{9},\sigma_{17},\\ \sigma_{19},\sigma_{20},\sigma_{21},\\ \sigma_{27},\sigma_{29},\sigma_{30},\\ \sigma_{31},\sigma_{32} \end{array}$

## Data Availability

Not applicable.
